# Reproductive period and epigenetic modifications of the oxidative phosphorylation pathway in the human prefrontal cortex

**DOI:** 10.1371/journal.pone.0199073

**Published:** 2018-07-27

**Authors:** Riley M. Bove, Ellis Patrick, Cristin McCabe Aubin, Gyan Srivastava, Julie A. Schneider, David A. Bennett, Philip L. De Jager, Lori B. Chibnik

**Affiliations:** 1 Weill Institute for the Neurosciences, Department of Neurology, University of California, San Francisco, San Francisco, CA, United States of America; 2 Harvard Medical School, Boston, MA, United States of America; 3 Program in Translational NeuroPsychiatric Genomics, Institute for the Neurosciences, Departments of Neurology and Psychiatry, Brigham and Women's Hospital, Boston, MA, United States of America; 4 Program in Medical and Population Genetics, Broad Institute, Cambridge, MA, United States of America; 5 School of Mathematics and Statistics, University of Sydney, Westmead, NSW, Australia; 6 The Westmead Institute for Medical Research, The University of Sydney, Westmead, NSW, Australia; 7 Rush Alzheimer's Disease Center, Rush University Medical Center, Chicago, IL, United States of America; 8 Center for Translational & Computational Neuroimmunology, Department of Neurology, Columbia University Medical Center, New York City, NY, United States of America; Western University of Health Sciences, UNITED STATES

## Abstract

**Purpose:**

Human females have a unique duration of post-reproductive longevity, during which sex-specific mechanisms ma influence later-life mechanisms of neuronal resilience and vulnerability. The maintenance of energy metabolism, through the oxidative phosphorylation (OXPHOS) apparatus, is essential for brain health. Given the known association between reproductive period (years from menarche to menopause) and cognitive aging, we examined the hypothesis that cumulative estrogen exposure across the lifetime may be associated with differential methylation of genes in the OXPHOS pathway.

**Methods:**

Using DNA methylation patterns in the post-mortem dorsolateral prefrontal cortex (DLPFC) of 426 women prospectively followed until death in the Religious Orders Study and Rush Memory and Aging Project, we examined the relationship between reproductive period (subtracting age at menarche from age at menopause) and DNA methylation of a published set of autosomal OXPHOS genes previously implicated in stroke susceptibility. We then performed an unsupervised analysis of methylation levels across the Hallmark pathways from the Molecular Signatures Database.

**Results:**

We observed a strong association between reproductive period and DNA methylation status across OXPHOS CpGs. We replicated this association between reproductive period and DNA methylation in a much larger set of OXPHOS genes in our unsupervised analysis. Here, reproductive period also showed associations with methylation in genes related to *E2F*, *MYC and MTORC1* signaling, fatty acid metabolism and DNA repair.

**Conclusion:**

This study provides evidence from both a supervised and unsupervised analyses, that lifetime cumulative endogenous steroid exposures may play a role in maintenance of post-menopausal cellular balance, including in brain tissue.

## Introduction

The maintenance of energy metabolism through the oxidative phosphorylation (OXPHOS) apparatus is essential for brain health [1–5]. Underscoring this essential role, mitochondrial injury and oxidation are part of the shared neuropathological mechanisms in neurological disorders including multiple sclerosis (MS), Alzheimer’s disease (AD), and Parkinson’s disease [1–4]. Furthermore, mutations of OXPHOS genes (the majority of which are encoded within the autosomal, not mitochondrial, genome [6]) are implicated in both rare [7] and common neurological disorders (e.g. stroke and neurodegeneration [5]), and in neuronal recovery after oxidative stress [8]. Little is known about the role of epigenetic modifications of the OXPHOS pathways in conferring resilience from or susceptibility to neurologic disease.

In an aging population, sex-specific mechanisms (including exposure to neuromodulatory gonadal steroids [9–11]) may influence the risk and progression of neurological diseases [12, 13]. Earlier menopause, and shorter reproductive period (years from menarche to menopause), have been associated with longitudinal cognitive decline, dementia (including AD), and neuropathology [14–19]. We hypothesize that endogenous hormonal exposures might modulate mechanisms of neurodegeneration shared between many diseases, such as OXPHOS [1, 20].

To examine the hypothesis that methylation of genes in the OXPHOS pathway is associated with lifetime estrogen exposure, we leveraged a rich data set of 426 female dorsolateral prefrontal cortex samples, from two well-phenotyped longitudinal cohorts.

## Materials and methods

### Participants

We examined DNA methylation patterns in the post-mortem dorsolateral prefrontal cortex (DLPFC) of 456 women who were enrolled in 2 prospectively followed cohorts maintained by investigators by the Rush Alzheimer’s Disease Center in Chicago, IL: the *Religious Orders Study* (ROS) and the *Rush Memory and Aging Project* (MAP)[21, 22]. The ROS cohort, established in 1994, consists of more than 1,400 older Catholic priests, nuns, and brothers from more than 40 groups in 13 states who were free of known dementia at the time of enrollment. The MAP cohort, established in 1997, consists of more than 1925 older men and women primarily from retirement facilities in the Chicago area who were free of known dementia at the time of enrollment. All participants in ROS and MAP sign an informed consent agreeing to annual detailed clinical evaluations and cognitive tests, and the rate of follow-up exceeds 90%. Similarly, participants in both cohorts signed an Anatomical Gift Act donating their brains at the time of death. The overall autopsy rate exceeds 85%. For the current analyses, we included 426 women with existing clinical, neuropathologic and brain methylation data. The DLPFC was selected as the initial region of interest, given its role in regulating executive function, an important outcome in these cohorts established to evaluate cognitive aging.

As in previous manuscripts, we analyzed the ROS and MAP cohorts jointly since they were designed to be combined, are collected by a single investigative team, and a large set of phenotypes collected are identical in both studies. All aspects of these studies were approved by the Institutional Review Boards of Rush University Medical Center and Partners Healthcare. More detailed information regarding the two cohorts can be found in previously published literature [21, 22].

### Hormonal variables

At baseline, participants were asked about current and past estrogen-based hormone therapy (HT) use (ever vs. never), age at which they started and stopped taking estrogen, age at menarche and menopause (final menstrual period), and whether menopause had occurred naturally or been induced surgically. We excluded women with other types of menopause. Current HT use was verified by inventory of prescription bottles that participants brought to the interview, with an agreement of 93%. As previously, data from 10 women were excluded because they provided highly unlikely ages at menopause (<20 or >60 years and 4 were excluded due to unlikely ages at menarche (>30) [14]. Reproductive period was calculated by subtracting age at menarche from age at menopause, as per [19].

### Oxidative phosphorylation genes

We used a set of 95 autosomal genes encoding proteins directly involved in the OXPHOS respiratory chain, selected based on published criteria from a chemical dissection of mitochondrial function, and previously associated with risk of ischemic stroke and intracranial hemorrhage in a large stroke consortium [5].

### DNA methylation

Our methods for obtaining methylation measures at 415,848 discrete CpG dinucleotides in ROS/MAP subjects have been previously described [23]. Briefly, 100 mg sections of frozen dorsolateral prefrontal cortex (DLPFC) were obtained from each deceased participant. These sections were thawed on ice, and the gray matter was carefully dissected from the white matter. DNA extraction was performed using the Qiagen (cat: 51306) QIAamp DNA mini protocol. The Qubit 2.0 Fluorometer was used to quantitate the DNA. 16uL of DNA at a concentration of 50ng/uL as measured by PicoGreen, was used by the Broad Institute's Genomics Platform for data generation by the Illumina InfiniumHumanMethylation450 bead chip assay. The platform produces a data file by implementing the recommended procedures of the proprietary Illumina GenomeStudio software, which includes color channel normalization and background removal. All data generation was conducted by laboratory personnel who were blinded as to the clinical and neuropathological phenotypes of each subject. Since we dissected out the gray matter from each sample, we profiled a piece of tissue composed primarily of different neuronal populations and other parenchymal cells such as glia.

Previously described quality checks of the data included (1) using the detection p-value criteria recommended by Illumina (i.e. < 0.01) to ensure the use of good quality probes, and (2) removal of probes strongly predicted by Illumina and observed by our team to cross-hybridize with the sex chromosomes based on sequence alignment or of autosomal, polymorphic CpGs where methylation level of these CpGs could be affected by a subject’s genotype (i.e. in which a Single Nucleotide Polymorphic site (SNP) with a minor allele frequency (MAF) ≥0.01 exists within 10 base pairs upstream or downstream of the CpG site. Finally, we removed subjects with poor quality individual data, according to Principal Component Analysis (PCA) (where data were within ± 3 SD from mean of a PC for PC1, PC2 and PC3 (detailed in [23]), and to bisulfite conversion (BC) efficiency. This left 420,132 probes and 426 subjects from both the ROS and MAP studies for downstream analysis. SWAN (subset-quantile within array normalization [24]) was used to normalize between the Infinium I and II probe types and account for technical variation. Estimates of the proportion of neurons in each sample and the bisulfite conversion efficiency were included in the model to also account for technical differences between arrays as in previous analysis [23].

### Gene expression

Gene expression data exists for 335 subjects of the 426 subjects with DNA methylation data. Gene expression was measured using FPKM values obtained from Illumina HiSeq RNA sequencing as previously described [25].

### Data analysis and statistical modeling

For our analyses, we used the β-values reported by the Illumina platform for each probe as the methylation level measurement for the targeted CpG site, as previously described [23]. These β-values range from 0 (no methylation) to 1 (100% methylation). Any missing β-value was imputed using a K-nearest neighbor algorithm for k = 100. For annotation of the CpG probes, we used the hg19 human reference genome and the IlluminaHumanMethylation450kanno.ilmn12.hg19 annotation.

In order to examine the association between cumulative estrogen exposure and OXPHOS gene methylation we first used a series of linear models to test the association of each probe with reproductive period (age of menopause minus age of menarche) adjusting for age at death, study (ROS or MAP), experimental batch, bisulfite conversion efficiency, the proportion of neuronal cells in the sample[26], race, cigarette pack-years smoked, surgical menopause and hormonal treatment. We then defined a set of OXPHOS related probes by selecting the probes annotated by Illumina to be near a previously published set of OXPHOS genes associated with neurovascular disease and dementia [5]. A Wilcoxon-rank-sum test was then used to test if the previous linear models indicated that OXPHOS probes were more associated with reproductive period than all other probes that were also annotated by Illumina as being near genes. This same approach was then used in an unsupervised manner to determine which Hallmark pathways included in the Broad Institute Molecular Signatures Database (MSigDB [27]) were most associated with reproductive period.

In order to examine the links between OXPHOS gene expression and reproductive span, as with the methylation data, linear models were first used to assess the relationship between all 23,205 genes with average FPKM greater than 1 and reproductive span accounting for the same confounders as in the methylation models. A Wilcoxon-rank-sum test was then used to test if the OXPHOS genes were more associated with reproductive span than all other genes. To assess if OXPHOS probes were correlated with gene expression, for each gene the average correlation between the gene and all its annotated probes were calculated.

## Results

### Descriptive characteristics

The 426 women who were part of one of two prospective cohorts of aging and dementia the Religious Orders Study and the Rush Memory and Aging Project (ROSMAP), were free of known dementia at study enrollment and followed annually until brain donation at death. Their demographic characteristics at the time of autopsy are summarized in **Table 1**. The mean (SD) menopausal age for women with natural menopause was 48.2 (5.4) and for surgical menopause was 42.3 (7.1). We found a large diversity in the duration of reproductive period, defined as age at last period minus age at first period, a marker of long-term estradiol exposure; this ranged from 8 to 48 years (median = 35). Methylation levels from prefrontal cortex samples were measured using an Illumina infinium HumanMethylation450K BeadChip as previously described [23, 28, 29].

**Table 1:** Demographic and reproductive characteristics of 426 women followed longitudinally.

**Demographic Characteristics**	
Age at death, mean (sd)	89.2 (6.6)
Study (ROS, MAP)	(237, 189)
Race	
% Caucasian	97.7%
Ethnicity	
% Hispanic	1.9%
Education years, mean (sd)	16 (3.4)
Smoking	
Pack-years smoked, mean (sd)	4.45 (13.8)
% Ever smoked	18.8%
**Reproductive Exposures**	
% HT use	20.3%
Menopausal type (% natural menopause)	70.4%
Age at menarche, mean (sd)	13.1 (1.6)
Age at menopause, mean (sd)	46.5 (6.5)
Natural menopause (N = 300)	48.2 (5.4)
Surgical menopause (N = 126)	42.3 (7.1)
Reproductive period, years (min, max)	(8,48)
*APOE* haplotype (22, 23, 24, 33, 34, 44)	(3, 67, 7, 245, 95, 5)
Braak stage (I, II, III, IV, V, VI)	(3, 25, 42, 123, 128, 103, 2)

### Association between reproductive period and methylation

We tested our primary hypothesis that reproductive period was assocated with methylation changes in OXPHOS genes using two complementary approaches. **In our first approach, we performed hypothesis-driven analyses** starting with the list of 95 OXPHOS genes previously shown to be associated with neurovascular diseases. We examined the association between reproductive period and DNA methylation in the 1344 CpGs annotated by Illumina as being in the TSS, 5’ or 3’ UTR or gene body of the 95 genes [5]. Using a Wilcoxon-rank-sum test we observed an association between subjects’ reproductive period and methylation of the set of OXPHOS genes (p = 1.6×10^−4^; adjusted for age at death, study (ROS or MAP), experimental batch, bisulfite conversion efficiency, estimated proportion of neuronal cells in the sample, race, cigarette pack-years smoked, menopause type and hormone therapy use). This association was primarily driven by positive relationships between methylation of OXPHOS CpGs and reproductive period (**S1 Fig**). **Table 2** shows the OXPHOS CpGs associated with reproductive period with a nominal p-value less than 0.01, as well as according to more stringent FDR. The most differentially methylated OXPHOS probe lies in the transcription start site of *NDUFS8*.

**Table 2:** Top P-Values for OXPHOS CpGs showing differential methylation by reproductive period (Age Menopause–Age Menarche).

*Probe*	*Chr*	*Position*	*Direction*	*P*.*value*	*Genes*	*Location*
cg07693657	chr11	67797975	+	4.90E-05	*NDUFS8*	TSS200
cg09192760	chr19	54606228	+	0.00024	*NDUFA3*	Body
cg18650732	chr19	5904804	+	0.00026	*VMAC;NDUFA11*	TSS200;TSS1500
cg13240639	chr22	30162856	-	0.00096	*UCRC;ZMAT5*	TSS1500;5'UTR
cg13635462	chr11	77790613	+	0.0019	*NDUFC2*	Body
cg21241477	chr9	136223965	-	0.0031	*SURF1;SURF2*	TSS1500;Body
cg04638486	chr5	52856570	+	0.0033	*NDUFS4*	1stExon
cg07595554	chr1	17380588	+	0.0054	*SDHB*	5'UTR;1stExon
cg16506172	chr14	50778832	+	0.0068	*L2HGDH;ATP5S*	1stExon;TSS1500
cg12532791	chr11	111957842	+	0.0069	*TIMM8B;SDHD*	TSS1500;Body
cg24441121	chr2	98262430	+	0.0072	*COX5B*	TSS200
cg18209341	chr17	13973192	-	0.0075	*COX10*	Body
cg00324562	chr21	27106440	+	0.008	*ATP5J;GABPA*	5'UTR;Body;TSS1500
cg21384827	chr11	67798041	+	0.0081	*NDUFS8*	TSS200
cg18548413	chr17	47492526	+	0.0093	*PHB*	TSS1500
cg27413508	chr20	30225517	+	0.01	*COX4I2*	TSS200
cg20639400	chr19	55866246	-	0.01	*COX6B2*	TSS200

Association between CpG methylation status and reproductive period for the top 17 OXPHOS probes that had a nominal p-value less than 0.01. For each probe associated with reproductive period its chromosome (Chr), position on the chromosome (Position), direction of association, nominal p-value of association with reproductive period (P.value). Genes that are annotated as being related to probe and the location where the probes are located in respect to the corresponding gene (Location).

**Fig 1:**
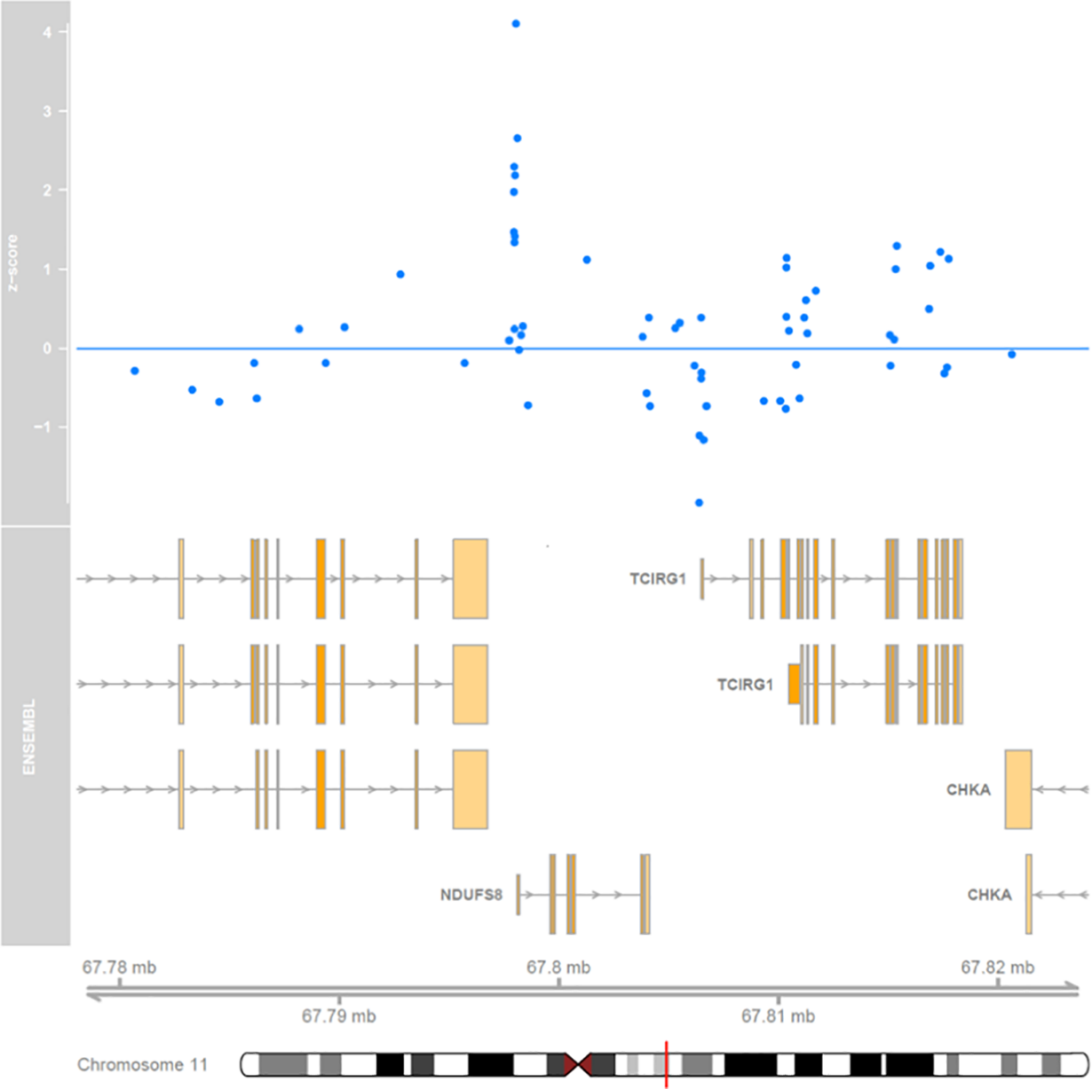
*NDUFS8* probes. The association of probes within ±20kb of *NDUFS8* with reproductive span are reported as z-scores. The locations of genes on chromosome 11 are given, along with their direction of transcription. Probes with a large positive z-score correspond to a large positive association with reproductive span and large negative z-score a large negative association. A z-score of ±1.96 corresponds to a two sided p-value of 0.05.

As an illustration, the methylation patterns of probes within ±20kb of *NDUFS8* are depicted in **Fig 1**.

Supporting a causal association between hormonal exposures and methylation changes, we noted an association between reproductive period and OXPHOS gene methylation levels in subjects with both natural (p = 4×10^−6^, n = 300) and surgical (p = 0.003, n = 126) menopause. HT use did not mitigate these associations.

**In our second approach,** using results from a series of Wilcoxon-rank-sum tests we performed an unsupervised analysis of the Hallmark pathways from the Molecular Signatures Database (MSigDB[27]) to find those most differentially methylated pathways in regards to reproductive period. The Hallmark oxidative phosphorylation pathway came up as the third most significant (p = 7.5×10^−6^) among 10 pathways that were differentially methylated with an FDR of 0.00012 (**Table 3**).

**Table 3:** Pathway analysis highlighting top pathways that show differential methylation by duration of reproductive period (Age Menopause–Age Menarche).

Hallmark Pathway	p-value	FDR	Top five genes
*E2F targets*	1.7×10^−11^	8.4×10^−10^	*PCNA*, *AK2*, *MRE11A*, *ANKRD49*, *AK2*
*MYC targets v1*	9.6×10^−11^	2.4×10^−9^	*PCNA*, *ABCE1*, *ANAPC10*, *DDX18*, *PRDX3*
*Oxidative phosphorylation*	7.5×10^−6^	0.00012	*NDUFS8*, *NDUFA3*, *PRDX3*, *PDHX*, *APIP*
*Fatty acid metabolism*	0.00021	0.0023	*ZSWIM3*, *ACOT8*, *BPHL*, *ADIPOR2*, *HADH*
*MTORC1 signaling*	0.00023	0.0023	*PRDX1*, *HMBS*, *GMPS*, *FKBP2*, *NAMPT*
*DNA repair*	0.00047	0.0039	*PCNA*, *RFC5*, *GTF2A2*, *SURF1*, *SURF2*
*MYC targets v2*	0.00059	0.0042	*DDX18*, *IMP4*, *CCDC115*, *MAP3K6*, *NOLC1*
*G2M checkpoint*	0.0012	0.0077	*STIL*, *PAFAH1B1*, *HOXC10*, *SYNCRIP*, *SAP30*
*Hypoxia*	0.0023	0.013	*HDLBP*, *SERPINE1*, *IGFBP3*, *KLF7*, *EFNA3*
*Heme metabolism*	0.0071	0.036	*ATP6V0A1*, *SNCA*, *TNRC6B*, *HMBS*, *AGPAT4*
*Adipogenesis*	0.015	0.0066	*NOTCH3*, *MAML2*, *SAP30*, *LFNG*, *SKP1*
*TNFA signaling via NFKB*	0.035	0.14	*AK2*, *PRDX3*, *AK2*, *IFNGR1*, *PRDX3*

An unsupervised pathway analysis was performed to determine which Hallmark pathways are associated with reproductive period. The p-value from a Wilcoxon-rank-sum test comparing the association with reproductive period of probes in a Hallmark pathway to all other probes is reported. The top five probes that are associated with reproductive period from each Hallmark pathway are also listed.

The Hallmark oxidative phosphorylation pathway largely overlaps with the set of 95 OXPHOS genes [5] used in our first approach, with 68 genes in common, 27 genes unique to the published OXPHOS set, and 133 genes unique to the Hallmark pathway. Underlying the strength of the association between reproductive period and OXPHOS, as it was one of the most epigenetically modified pathways. In addition to OXPHOS, other important pathways identified included pathways involved in cell cycle regulation (E2F, G2M checkpoint), DNA synthesis and repair (E2F, DNA repair), gene expression (MYC targets v1 and v2), synaptic plasticity (MTORC1 signaling), metabolism (fatty acid, heme), and, hypoxia.

### Functional analyses

The ROSMAP cohort has gene expression data for 335 of the women in our analyses [25]. Here, the average correlation between methylation level of OXPHOS probes and expression of their annotated gene was -0.039. However, while reproductive period was associated with methylation levels of OXPHOS CpGs, there was no such association at the gene expression level (Wilcoxon rank-sum test, p = 0.977). This suggests that while there is most likely a regulatory relationship between the OXPHOS CpGs and their genes, this relationship is not seen with respect to reproductive period.

## Discussion

Using both a hypothesis-driven and an unsupervised pathway analysis approach, we found that longer reproductive period was significantly associated with subsequent levels of methylation across a set of OXPHOS genes, genes known to be associated with stroke, a major cause of worldwide mortality and neurological morbidity.

It is known that sex influences epigenetic modifications, including DNA methylation, in the blood and across many tissues[30], potentially influencing risk for a number of diseases (e.g. common cancers and neuropsychiatric diseases [31, 32]). In the brain, sex drives neural development, both perinatally and subsequently, leading to permanent alteration of some neural networks and thus sexually dimorphic brain regions. The brain is under complex transcriptional regulation [33–41], which proceeds according to a globally similar specific spatial and temporal architecture [41]; the extent of sexual dimorphism in gene expression is unique to primate species [42], is region-specific [43], and is present across a number of important regulatory pathways and over time [43, 44]. Some of these differences may be modulated by sex hormone receptors [45], and epigenetic regulation of gonadal hormone receptor promoters occurs across development in sexually dimorphic brain areas. Consequently, hormonal and non-hormonal responses are altered throughout the lifespan to modulate specific brain functions and neuronal connectivity. In neuropsychiatric diseases, particularly diseases that show sexual dimorphism in epidemiology, some studies have revealed sexually dimorphic patterns of epigenetic phenomena [46] (e.g. schizophrenia and bipolar disorder [47–49]).

This study had three important limitations. First, given their complexity and potential for inactivation and dosing effects, the sex chromosomes were not included in the current methylation analyses; however, OXPHOS genes are found primarily on autosomes. Second, we used only an estimate of the proportion of neuronal cells in the sample, and methylation levels across other cell types might have been confounders. Third, the lack of association between reproductive exposures and RNA expression suggests that other variables in the postmenopausal period may play a stronger role in regulating expression. Finally, it is important to note that the study did not evaluate for other possible modifiers of DNA methylation, such as smoking or medications.

## Conclusion

Human females are unique among mammals in the duration of post-reproductive longevity. Previous investigations of sex-specific mechanisms leading to neurological resilience have uncovered an association between reproductive period, i.e. a woman’s period of maximal exposure to levels of estradiol and other gonadal hormones, and cognitive decline in old age. Here, we advanced these findings mechanistically with reporting a significant association of reproductive period with methylation levels across genes in the OXPHOS pathway. This provides some mechanistic suggestion that early and mid-life hormonal exposures may influence risk of late-life neurological morbidity and mortality via epigenetic regulation of energy metabolism.

## Supporting information

S1 Fig Barcode plot of OXPHOS probes. A plot demonstrating the ranks of each of the 1,344 OXPHOS probes with respect to all 420,132 included probes. Each black vertical line represents an OXPHOS probe. A centered cumulative sum is also plotted as a blue line. Tracking the ranks of probes from left to right, when the blue line increases the number of OXPHOS probes seen is increasing more than expected and vice versa for when the blue line decreases.
(PNG)

## References

[1] Lassmann H. Mechanisms of neurodegeneration shared between multiple sclerosis and Alzheimer's disease. J Neural Transm. 2011;118(5):747–52. Epub 2011/03/05. 10.1007/s00702-011-0607-8 . 21373761

[2] Muller WE, Eckert A, Kurz C, Eckert GP, Leuner K. Mitochondrial dysfunction: common final pathway in brain aging and Alzheimer's disease—therapeutic aspects. Molecular neurobiology. 2010;41(2–3):159–71. Epub 2010/05/13. 10.1007/s12035-010-8141-5 . 20461558

[3] Lopez-Gallardo E, Iceta R, Iglesias E, Montoya J, Ruiz-Pesini E. OXPHOS toxicogenomics and Parkinson's disease. Mutat Res. 2011;728(3):98–106. Epub 2011/07/19. 10.1016/j.mrrev.2011.06.004 . 21763451

[4] Coskun P, Wyrembak J, Schriner SE, Chen HW, Marciniack C, Laferla F, et al A mitochondrial etiology of Alzheimer and Parkinson disease. Biochimica et biophysica acta. 2012;1820(5):553–64. Epub 2011/08/30. 10.1016/j.bbagen.2011.08.008 ; PubMed Central PMCID: PMC3270155. 21871538 PMC3270155

[5] Anderson CD, Biffi A, Nalls MA, Devan WJ, Schwab K, Ayres AM, et al Common variants within oxidative phosphorylation genes influence risk of ischemic stroke and intracerebral hemorrhage. Stroke. 2013;44(3):612–9. Epub 2013/01/31. 10.1161/STROKEAHA.112.672089 ; PubMed Central PMCID: PMC3582722. 23362085 PMC3582722

[6] Wagner BK, Kitami T, Gilbert TJ, Peck D, Ramanathan A, Schreiber SL, et al Large-scale chemical dissection of mitochondrial function. Nat Biotechnol. 2008;26(3):343–51. Epub 2008/02/26. 10.1038/nbt1387 ; PubMed Central PMCID: PMC2715872. 18297058 PMC2715872

[7] DiMauro S, Schon EA. Mitochondrial disorders in the nervous system. Annual review of neuroscience. 2008;31:91–123. Epub 2008/03/13. 10.1146/annurev.neuro.30.051606.094302 . 18333761

[8] Nicholls DG. Oxidative stress and energy crises in neuronal dysfunction. Annals of the New York Academy of Sciences. 2008;1147:53–60. Epub 2008/12/17. 10.1196/annals.1427.002 . 19076430

[9] Barron AM, Pike CJ. Sex hormones, aging, and Alzheimer's disease. Front Biosci (Elite Ed). 2012;4:976–97. Epub 2011/12/29. . 22201929 10.2741/e434 PMC3511049

[10] Boulware MI, Kent BA, Frick KM. The impact of age-related ovarian hormone loss on cognitive and neural function. Curr Top Behav Neurosci. 2012;10:165–84. Epub 2011/05/03. 10.1007/7854_2011_122 . 21533680

[11] Spence RD, Hamby ME, Umeda E, Itoh N, Du S, Wisdom AJ, et al Neuroprotection mediated through estrogen receptor-alpha in astrocytes. Proceedings of the National Academy of Sciences of the United States of America. 2011;108(21):8867–72. Epub 2011/05/11. 10.1073/pnas.1103833108 ; PubMed Central PMCID: PMC3102368. 21555578 PMC3102368

[12] Cahill L. Why sex matters for neuroscience. Nature reviews Neuroscience. 2006;7(6):477–84. Epub 2006/05/12. 10.1038/nrn1909 . 16688123

[13] Clayton JA, Collins FS. Policy: NIH to balance sex in cell and animal studies. Nature. 2014;509(7500):282–3. Epub 2014/05/17. . 24834516 10.1038/509282a PMC5101948

[14] Bove R, Secor E, Chibnik LB, Barnes LL, Schneider JA, Bennett DA, et al Age at surgical menopause influences cognitive decline and Alzheimer pathology in older women. Neurology. 2014;82(3):222–9. 10.1212/WNL.0000000000000033 ; PubMed Central PMCID: PMCPMC3902759. 24336141 PMC3902759

[15] Corbo RM, Gambina G, Broggio E, Scacchi R. Influence of Variation in the Follicle-Stimulating Hormone Receptor Gene (FSHR) and Age at Menopause on the Development of Alzheimer's Disease in Women. Dement Geriatr Cogn Disord. 2011;32(1):63–9. Epub 2011/08/26. 10.1159/000330472 . 21865747

[16] Coppus AM, Evenhuis HM, Verberne GJ, Visser FE, Eikelenboom P, van Gool WA, et al Early age at menopause is associated with increased risk of dementia and mortality in women with Down syndrome. J Alzheimers Dis. 2010;19(2):545–50. Epub 2010/01/30. 10.3233/JAD-2010-1247 . 20110600

[17] Vearncombe KJ, Pachana NA. Is cognitive functioning detrimentally affected after early, induced menopause? Menopause. 2009;16(1):188–98. Epub 2008/08/30. 10.1097/gme.0b013e3181775eb4 . 18724262

[18] Henderson VW, Sherwin BB. Surgical versus natural menopause: cognitive issues. Menopause. 2007;14(3 Pt 2):572–9. Epub 2007/05/04. 10.1097/gme.0b013e31803df49c . 17476147

[19] Geerlings MI, Ruitenberg A, Witteman JC, van Swieten JC, Hofman A, van Duijn CM, et al Reproductive period and risk of dementia in postmenopausal women. Jama. 2001;285(11):1475–81. Epub 2001/03/23. . 11255424 10.1001/jama.285.11.1475

[20] Federico A, Cardaioli E, Da Pozzo P, Formichi P, Gallus GN, Radi E. Mitochondria, oxidative stress and neurodegeneration. J Neurol Sci. 2012;322(1–2):254–62. 10.1016/j.jns.2012.05.030 . 22669122

[21] Bennett DA, Schneider JA, Arvanitakis Z, Wilson RS. Overview and findings from the religious orders study. Current Alzheimer research. 2012;9(6):628–45. 22471860 10.2174/156720512801322573 PMC3409291

[22] Bennett DA, Schneider JA, Buchman AS, Barnes LL, Boyle PA, Wilson RS. Overview and findings from the rush Memory and Aging Project. Current Alzheimer research. 2012;9(6):646–63. 22471867 10.2174/156720512801322663 PMC3439198

[23] De Jager PL, Srivastava G, Lunnon K, Burgess J, Schalkwyk LC, Yu L, et al Alzheimer's disease: early alterations in brain DNA methylation at ANK1, BIN1, RHBDF2 and other loci. Nature neuroscience. 2014;17(9):1156–63. Epub 2014/08/19. 10.1038/nn.3786 ; PubMed Central PMCID: PMC4292795. 25129075 PMC4292795

[24] Maksimovic J, Gordon L, Oshlack A. SWAN: Subset-quantile within array normalization for illumina infinium HumanMethylation450 BeadChips. Genome biology. 2012;13(6):R44 Epub 2012/06/19. 10.1186/gb-2012-13-6-r44 ; PubMed Central PMCID: PMC3446316. 22703947 PMC3446316

[25] Lim AS, Srivastava GP, Yu L, Chibnik LB, Xu J, Buchman AS, et al 24-hour rhythms of DNA methylation and their relation with rhythms of RNA expression in the human dorsolateral prefrontal cortex. PLoS genetics. 2014;10(11). 10.1371/journal.pgen.1004792 25375876 PMC4222754

[26] De Jager PL, Srivastava G, Lunnon K, Burgess J, Schalkwyk LC, Yu L, et al Alzheimer's disease: early alterations in brain DNA methylation at ANK1, BIN1, RHBDF2 and other loci. Nature neuroscience. 2014;17(9):1156–63. 10.1038/nn.3786 25129075 PMC4292795

[27] Subramanian A, Tamayo P, Mootha VK, Mukherjee S, Ebert BL, Gillette MA, et al Gene set enrichment analysis: a knowledge-based approach for interpreting genome-wide expression profiles. Proceedings of the National Academy of Sciences of the United States of America. 2005;102(43):15545–50. Epub 2005/10/04. 10.1073/pnas.0506580102 ; PubMed Central PMCID: PMC1239896. 16199517 PMC1239896

[28] Yu L, Chibnik L, Yang J, McCabe C, Xu J, Schneider JA, et al Methylation profiles in peripheral blood CD4+ lymphocytes versus brain: The relation to Alzheimer's disease pathology. Alzheimers Dement. 2016 10.1016/j.jalz.2016.02.009 . 27016692 PMC5014706

[29] Yang J, Yu L, Gaiteri C, Srivastava GP, Chibnik LB, Leurgans SE, et al Association of DNA methylation in the brain with age in older persons is confounded by common neuropathologies. Int J Biochem Cell Biol. 2015;67:58–64. 10.1016/j.biocel.2015.05.009 ; PubMed Central PMCID: PMCPMC4564337. 26003740 PMC4564337

[30] Zhu ZZ, Hou L, Bollati V, Tarantini L, Marinelli B, Cantone L, et al Predictors of global methylation levels in blood DNA of healthy subjects: a combined analysis. Int J Epidemiol. 2010 Epub 2010/09/18. 10.1093/ije/dyq154 . 20846947 PMC3304518

[31] Kirsch-Volders M, Bonassi S, Herceg Z, Hirvonen A, Moller L, Phillips DH. Gender-related differences in response to mutagens and carcinogens. Mutagenesis. 2010;25(3):213–21. Epub 2010/03/03. 10.1093/mutage/geq008 . 20194421

[32] Pregelj P. Neurobiological aspects of psychosis and gender. Psychiatr Danub. 2009;21 Suppl 1:128–31. Epub 2009/12/03. . 19789497

[33] Heinzen EL, Ge D, Cronin KD, Maia JM, Shianna KV, Gabriel WN, et al Tissue-specific genetic control of splicing: implications for the study of complex traits. PLoS Biol. 2008;6(12):e1 Epub 2009/02/19. 10.1371/journal.pbio.1000001 ; PubMed Central PMCID: PMC2605930. 19222302 PMC2605930

[34] Gibbs JR, van der Brug MP, Hernandez DG, Traynor BJ, Nalls MA, Lai SL, et al Abundant quantitative trait loci exist for DNA methylation and gene expression in human brain. PLoS genetics. 2010;6(5):e1000952 Epub 2010/05/21. 10.1371/journal.pgen.1000952 ; PubMed Central PMCID: PMC2869317. 20485568 PMC2869317

[35] Liu C, Cheng L, Badner JA, Zhang D, Craig DW, Redman M, et al Whole-genome association mapping of gene expression in the human prefrontal cortex. Mol Psychiatry. 2010;15(8):779–84. Epub 2010/03/31. 10.1038/mp.2009.128 ; PubMed Central PMCID: PMC3057235. 20351726 PMC3057235

[36] Johnson MB, Kawasawa YI, Mason CE, Krsnik Z, Coppola G, Bogdanovic D, et al Functional and evolutionary insights into human brain development through global transcriptome analysis. Neuron. 2009;62(4):494–509. Epub 2009/05/30. 10.1016/j.neuron.2009.03.027 ; PubMed Central PMCID: PMC2739738. 19477152 PMC2739738

[37] Somel M, Guo S, Fu N, Yan Z, Hu HY, Xu Y, et al MicroRNA, mRNA, and protein expression link development and aging in human and macaque brain. Genome Res. 2010;20(9):1207–18. Epub 2010/07/22. 10.1101/gr.106849.110 ; PubMed Central PMCID: PMC2928499. 20647238 PMC2928499

[38] Oldham MC, Konopka G, Iwamoto K, Langfelder P, Kato T, Horvath S, et al Functional organization of the transcriptome in human brain. Nature neuroscience. 2008;11(11):1271–82. Epub 2008/10/14. 10.1038/nn.2207 ; PubMed Central PMCID: PMC2756411. 18849986 PMC2756411

[39] Myers AJ, Gibbs JR, Webster JA, Rohrer K, Zhao A, Marlowe L, et al A survey of genetic human cortical gene expression. Nat Genet. 2007;39(12):1494–9. Epub 2007/11/06. 10.1038/ng.2007.16 . 17982457

[40] Webster JA, Gibbs JR, Clarke J, Ray M, Zhang W, Holmans P, et al Genetic control of human brain transcript expression in Alzheimer disease. Am J Hum Genet. 2009;84(4):445–58. Epub 2009/04/14. 10.1016/j.ajhg.2009.03.011 ; PubMed Central PMCID: PMC2667989. 19361613 PMC2667989

[41] Colantuoni C, Lipska BK, Ye T, Hyde TM, Tao R, Leek JT, et al Temporal dynamics and genetic control of transcription in the human prefrontal cortex. Nature. 2011;478(7370):519–23. Epub 2011/10/28. 10.1038/nature10524 . 22031444 PMC3510670

[42] Reinius B, Saetre P, Leonard JA, Blekhman R, Merino-Martinez R, Gilad Y, et al An evolutionarily conserved sexual signature in the primate brain. PLoS genetics. 2008;4(6):e1000100 Epub 2008/06/21. 10.1371/journal.pgen.1000100 ; PubMed Central PMCID: PMC2413013. 18566661 PMC2413013

[43] Weickert CS, Elashoff M, Richards AB, Sinclair D, Bahn S, Paabo S, et al Transcriptome analysis of male-female differences in prefrontal cortical development. Mol Psychiatry. 2009;14(6):558–61. Epub 2009/05/21. 10.1038/mp.2009.5 . 19455171

[44] Berchtold NC, Cribbs DH, Coleman PD, Rogers J, Head E, Kim R, et al Gene expression changes in the course of normal brain aging are sexually dimorphic. Proceedings of the National Academy of Sciences of the United States of America. 2008;105(40):15605–10. Epub 2008/10/04. 10.1073/pnas.0806883105 ; PubMed Central PMCID: PMC2563070. 18832152 PMC2563070

[45] Lai JC, Cheng YW, Chiou HL, Wu MF, Chen CY, Lee H. Gender difference in estrogen receptor alpha promoter hypermethylation and its prognostic value in non-small cell lung cancer. Int J Cancer. 2005;117(6):974–80. Epub 2005/06/30. 10.1002/ijc.21278 . 15986439

[46] Qureshi IA, Mehler MF. Genetic and epigenetic underpinnings of sex differences in the brain and in neurological and psychiatric disease susceptibility. Prog Brain Res. 2010;186:77–95. Epub 2010/11/26. 10.1016/B978-0-444-53630-3.00006-3 . 21094887 PMC4465286

[47] Carrard A, Salzmann A, Malafosse A, Karege F. Increased DNA methylation status of the serotonin receptor 5HTR1A gene promoter in schizophrenia and bipolar disorder. J Affect Disord. 2011;132(3):450–3. Epub 2011/04/02. 10.1016/j.jad.2011.03.018 . 21453976

[48] Connor CM, Akbarian S. DNA methylation changes in schizophrenia and bipolar disorder. Epigenetics. 2008;3(2):55–8. Epub 2008/04/10. . 18398310 10.4161/epi.3.2.5938

[49] Mill J, Tang T, Kaminsky Z, Khare T, Yazdanpanah S, Bouchard L, et al Epigenomic profiling reveals DNA-methylation changes associated with major psychosis. Am J Hum Genet. 2008;82(3):696–711. Epub 2008/03/06. 10.1016/j.ajhg.2008.01.008 ; PubMed Central PMCID: PMC2427301. 18319075 PMC2427301

