# Supplementary figures and images for: Reproductive period and epigenetic modifications of the oxidative phosphorylation pathway in the human prefrontal cortex
Source: PLoS One. 2018 Jul 27;13(7):e0199073. doi: 10.1371/journal.pone.0199073 (PMC6063396; doi:10.1371/journal.pone.0199073)

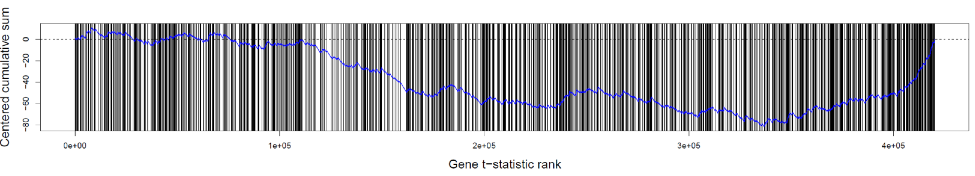

Supplement: S1 Fig — A plot demonstrating the ranks of each of the 1,344 OXPHOS probes with respect to all 420,132 included probes. Each black vertical line represents an OXPHOS probe. A centered cumulative sum is also plotted as a blue line. Tracking the ranks of probes from left to right, when the blue line increases the number of OXPHOS probes seen is increasing more than expected and vice versa for when the blue line decreases. (PNG) [file pone.0199073.s001.png]
